# The applicability of compost, zeolite and calcium oxide in assisted remediation of acidic soil contaminated with Cr(III) and Cr(VI)

**DOI:** 10.1007/s11356-019-05221-y

**Published:** 2019-05-23

**Authors:** Maja Radziemska, Mirosław Wyszkowski, Agnieszka Bęś, Zbigniew Mazur, Jerzy Jeznach, Martin Brtnický

**Affiliations:** 1 Department of Environmental Improvement, Faculty of Civil and Environmental Engineering, Warsaw University of Life Sciences – SGGW, Nowoursynowska 159, 02-776 Warsaw, Poland; 2 Department of Environmental Chemistry, Faculty of Environmental Management and Agriculture, University of Warmia and Mazury in Olsztyn, Pl. Łódzki 4, 10-727 Olsztyn, Poland; 3 Department of Chemistry, Research Group of Environmental Toxicology, University of Warmia and Mazury in Olsztyn, Prawocheńskiego 17, 10-720 Olsztyn, Poland; 4 Department of Geology and Pedology, Faculty of Forestry and Wood Technology, Mendel University in Brno, Zemědělská 1/1665, 613 00 Brno, Czech Republic; 5 Central European Institute of Technology, Brno University of Technology, Purkyňova 656/123, 612 00 Brno, Czech Republic

**Keywords:** Chromium contamination, Soil amendments, Compost, Zeolite, Calcium oxide

## Abstract

The effect of soil amendments, i.e., compost, zeolite, and calcium oxide, on the chemical properties of soil contaminated with Cr(III) and Cr(VI) and the uptake of selected heavy metals by spring barley (*Hordeum vulgare* L.) and maize (*Zea mays* L.) was determined in a pot experiment. The content of all investigated heavy metals in the tested plants varied significantly in response to the tested soil amendments and increasing concentrations of Cr(III) and Cr(VI). Compost, zeolite, and calcium oxide contributed to an increase in the average yield of the aerial parts of maize plants only in treatments contaminated with Cr(III). The concentrations of Cr, Zn, and Ni in the aerial parts of spring barley and maize were higher in treatments contaminated with Cr(III) than in treatments contaminated with Cr(VI). Calcium oxide induced a significant increase in soil pH relative to the control treatment. In treatments without soil amendments, the average Cr content of soil was higher in pots contaminated with Cr(VI). The concentrations of Zn and Cu in non-amended treatments were negatively correlated with increasing doses of Cr(III) and Cr(VI). Calcium oxide decreased the average content of Cr, Cu, and Ni in all experimental variants. Compost increased the average content of Zn in treatments contaminated with Cr(III) and Cr(IV) relative to non-amended soil.

## Introduction

Human activities significantly contribute to land transformation (Kintl et al. 2018; Krzyżaniak et al. 2019). In many cases, the resulting changes are immense, and they lead to the degradation of land (Kust et al. 2018; Majewski et al. 2011). Low levels of knowledge about the available land reclamation options are one of the main barriers to recovering disturbed land to its former or other productive uses. Heavy-metal contamination poses a serious environmental threat around the world (Arfaeinia et al. 2019). According to the European Environment Agency, more than 35% of land in Europe is contaminated with heavy metals (Radziemska et al. 2017a). Chromium (Cr) is a trace element and the twentieth most abundant element on the planet that accounts for 0.037% of the Earth’s crust. It is also the fourth most abundant element in the group of 29 elements which are regarded as critical on account of their biological role and toxicity. Chromium occurs naturally, but it is also released into the environment from anthropogenic sources (Raptis et al. 2018). According to the literature, Cr compounds in the natural environment can interact with mineral and organic compounds (Hu et al. 2018). Some of these compounds are soluble in water, they are readily transported by surface and underground water, and they are easily dispersed in the natural environment (Wyszkowski and Radziemska 2013).

Naturally occurring Cr compounds are characterized by various degrees of toxicity. In the environment, Cr(VI) compounds are easily reduced by organic matter to Cr(III) (Lilli et al. 2019). The biological activity of chromium is determined by its valence state. Chromium compounds are stable in trivalent (Cr(III)) and hexavalent (Cr(VI)) states (Jobby et al. 2018). Trivalent chromium compounds are approximately one hundred times less toxic than hexavalent compounds, and only Cr(VI) compounds are mutagenic (Goswami et al. 2018). In addition to differences in their biological and toxicological properties, the mobility of Cr compounds in soil and their uptake by plants also differ subject to soil microbiological activity and the organic matter content of soil (Gupta et al. 2018). In most cases, soil reclamation by physical and chemical means is not economically justified, but contaminated soils pose an environmental hazard and cannot be left untreated (He et al. 2019). Phytoremediation (“green remediation”) techniques offer an effective alternative. This biological method relies on living plants which degrade (phytodegradation), immobilize (phytostabilization), and remove pollutants from soil (phytoextraction) (Radziemska et al. 2017b).

The application of phytoremediation methods in contaminated areas is a long process because plants differ in their ability to extract and stabilize contaminants (Cameselle and Gouveia 2019). Therefore, soil amendments can be used to increase the effectiveness of phytoremediation and achieve the desired results. The most popular soil amendments include sawdust, biocompost, peat, brown coal, zeolite, phosphorites, apatites, phosphate fertilizers, calcium carbonate, dolomite, and volatile ashes (Ahmad et al. 2012; Li et al. 2019; He et al. 2018; Radziemska and Mazur 2016).

The aim of this study was to determine the applicability of soil amendments such as compost, zeolite, and CaO, in assisted remediation of soil contaminated with Cr(III) and Cr(VI) compounds and sown with spring barley (*Hordeum vulgare* L.) and maize (*Zea mays* L.).

## Materials and methods

### Soil and amendments

Acidic soil was collected from the top layer (0–20 cm) of a non-contaminated site in an agricultural area in the vicinity of Olsztyn, Poland (53° 35′ 45″ N, 19° 51′ 06″ E). The physicochemical properties of soil samples are presented in Table 1.

**Table 1:** Selected physicochemical properties of the studied soil

Property	Unit	Value
pH	–	4.83
Cation exchange capacity	cmol kg^−1^	33.75
Texture	–	Fine sand^c^
Total organic carbon	g kg^−1^	7.13
Phosphorous	mg kg^−1^	46.61
Potassium	mg kg^−1^	8.22
Magnesium	mg kg^−1^	33.91
Chromium	mg kg^−1^	12.95
Copper	mg kg^−1^	9.01
Zinc	mg kg^−1^	24.25
Nickel	mg kg^−1^	3.99

^c^According to USDA

The chemical composition of soil amendments used in the experiment is presented in Table 2. Compost and zeolite were added to soil in amounts corresponding to 2.0% (each) of soil dry mass, and 50% calcium oxide was added in the amount of 1.25 g kg^−1^ of soil, i.e., a dose corresponding to one unit of hydrolytic acidity (HAC). Soil samples were thoroughly mixed and allowed to stabilize under natural conditions over a period of three weeks before the greenhouse experiment.

**Table 2:** Chemical composition of soil amendments used in the experiment

Property	Unit	Compost	Zeolite	Calcium oxide
Phosphorous	g kg^−1^	2.32	0.11	0.10
Potassium	g kg^−1^	1.33	23.21	0.77
Magnesium	g kg^−1^	1.47	0.31	2.65
Chromium	mg kg^−1^	3.48	1.81	2.70
Copper	mg kg^−1^	38.13	12.38 s	2.26
Zinc	mg kg^−1^	31.80	14.68	5.14
Nickel	mg kg^−1^	18.75	408.7	6.64

### Greenhouse experiment

The pot experiment was carried out in the plant growth facility of the University of Warmia and Mazury in Olsztyn. The effect of Cr(III) and Cr(IV) as well as compost, zeolite, and calcium oxide on the content of selected heavy metals was evaluated in 9.5-kg polyethylene pots in a greenhouse experiment. The pots were exposed to natural daylight, temperature of 20–25 °C, and relative humidity of 60–70%. Soil was experimentally contaminated with aqueous solutions of Cr(III), applied in the form of KCr(SO_4_)_2_·12H_2_O, and Cr(VI), applied in the form of K_2_Cr_2_O_7_, at 0 (control), 25, 50, 100, and 150 mg kg^−1^ soil. Soil was also enhanced with the following macronutrients and micronutrients (in mg·kg^−l^ soil): N—110 [CO(NH_2_)_2_ + (NH_4_)_6_Mo_7_O_24_·4H_2_O + (NH_4_)_2_HPO_4_]; P—50 [(NH_4_)_2_HPO_4_]; K—110 [KCl + KCr(SO_4_)_2_·12H_2_O + K_2_Cr_2_O_7_]; Mg—50 [MgSO_4_·7H_2_O]; Mn—5 [MnCl_2_·4H_2_O]; Mo—5 [(NH_4_)_6_Mo_7_O_24_·4H_2_O]; B—0.33 [H_3_BO_3_]. The experiment had a completely randomized block design with four replicates per treatment. The tested plants were spring barley (*Hordeum vulgare* L.) cv. Ortega (main crop) and maize (*Zea mays* L.) cv. Fripon (successive crop). The main crop was sown at 15 plants per pot, and the successive crop at 8 plants per pot. The plants were irrigated with distilled water to maintain soil moisture content at 60% capillary water capacity. Spring barley was harvested in the heading stage, and maize was harvested in the stem elongation stage on days 56 and 67 of the growing season, respectively. The weight of aerial plant parts was determined at harvest. The harvested biomass was dried, ground, and subjected to chemical analyses.

### Sample preparation and chemical analysis

Soil samples were air-dried and sieved (< 1 mm). Plant material was washed with distilled water and oven-dried at 70 °C. Dried plant material was ground in an analytical mill (Retsch type ZM 300, Hann, Germany). The following soil parameters were determined before the experiment and at harvest: pH was determined in a 1:5 *w*/*v* suspension in distilled water with a pH meter (Model EA940, Orion, USA), total N was determined by the Kjeldahl (1983) method, total organic carbon (TOC) was determined after dichromate oxidation of samples and titration with ferrous ammonium sulfate (Walkley and Black 1934), available P was determined in a colorimetric analysis with the vanadium-molybdenum method (Cavell 1955), K was determined by atomic emission spectrometry (AES), and Mg was determined by atomic absorption spectrometry (AAS) (Szyszko 1982). Total Cr, Cu, Zn, and Ni content was determined with the SpectrAA 240FS (VARIAN, Australia) atomic absorption spectrophotometer after digestion of triplicate samples in HNO_3_ in the MARS 5 microwave oven (CEM Corporation, USA) with a modified version of the EPA 3051A method (U.S. Environmental Protection Agency 2007). The analyses were conducted with the use of deionized water with conductivity of 0.055 μS cm^−1^, purified in the Crystal 10 system (Aldrona Laboratory System). Certified reference material (Sigma Aldrich Chemie GmbH, No. BCR142R) was used in the analyses. Before the experiment, laboratory equipment was treated with 5 mol L^−1^ HNO_3_ for 24 h and rinsed with ultrapure water.

### Statistical analyses

The normal distribution of variables in every independent group was checked in the Shapiro-Wilk test, and the normality of the residuals was verified. The homogeneity of variance was checked in Levene’s test. Factorial ANOVA (*F*-test) was conducted. The experimental factors were chromium valence, chromium dose, and soil amendments. The significance of differences was determined in Tukey’s test (*p* < 0.05). The relationships between selected parameters were determined by calculating Pearson’s correlation coefficient *r* between two variables. The calculated values of *r* were regarded as significant at *p* < 0.05. The relationships between selected parameters were determined by correlation analysis and linear regression analysis with three variables. The results were processed in the Statistica 12.0 program (StatSoft Inc. 2017).

## Results

### The effect of Cr(III) and Cr(VI) contamination on dry biomass yield

**Fig 1:**
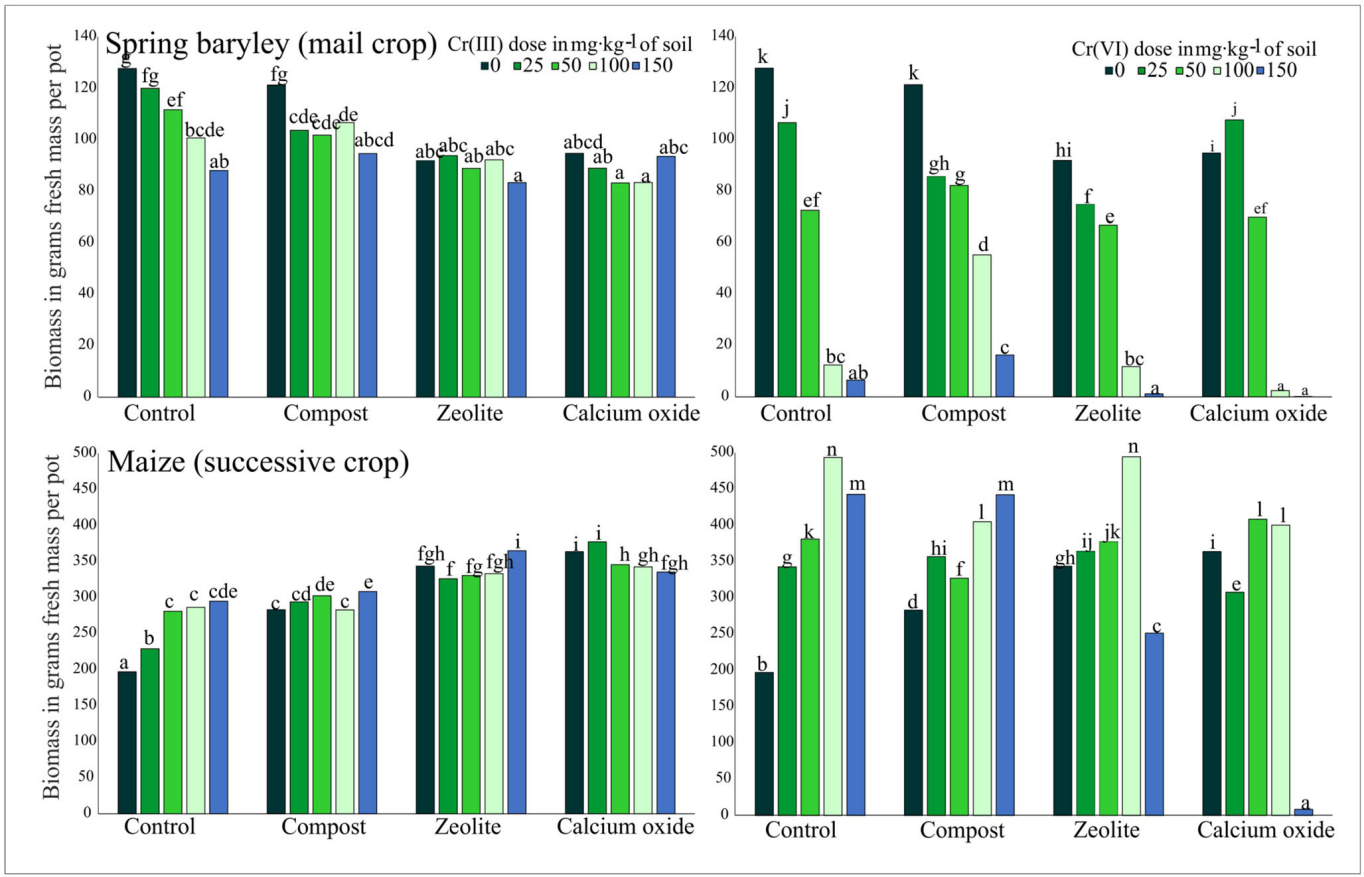
The biomass yields of spring barley (main crop) and maize (successive crop) in pots contaminated with different doses of Cr(III) and Cr(VI) (0; 25; 50; 150 mg kg^−1^ soil). Different letters above the columns indicated significant difference at the *p <* 0.05

Crop yields differed significantly in response to soil contamination with various forms of Cr (Tables 3 and 4). Spring barley (main crop) in treatments without soil amendments was highly sensitive to contamination with Cr(VI) (Fig. 1). The reverse was noted in maize (successive crop) where increasing doses of Cr(III) and, in particular, Cr(VI) induced a significant increase in yield. Cr(III) doses of 100 and 150 mg kg^−1^ soil decreased the weight of the aerial parts of spring barley by 21% and 31%, respectively, relative to the control treatment. The Cr(VI) dose of 150 mg kg^−1^ soil caused a 95% decrease in the above parameter. Maize yields were highest under exposure to a Cr(VI) dose of 100 mg kg^−1^ soil.

**Table 3:** Analysis of variance (*F*-test) of heavy-metal content in aboveground parts of spring barley

Source of variation	Cr		Cu		Zn		Ni		Biomass yield	
	*F*	*p* value	*F*	*p* value	*F*	*p* value	*F*	*p* value	*F*	*p* value
V	17711.8**	< 0.001	39393.4**	< 0.001	3296.7**	< 0.001	6509.5**	< 0.001	6634.4**	< 0.001
D	5335.4**	< 0.001	5866.9**	< 0.001	1973.0**	< 0.001	913.9**	< 0.001	2432.9**	< 0.001
SA	8994.0**	< 0.001	3961.3**	< 0.001	422.2**	< 0.001	2371.7**	< 0.001	463.6**	< 0.001
V×D	1369.4**	< 0.001	2672.0**	< 0.001	261.2**	< 0.001	468.6**	< 0.001	1375.5**	< 0.001
V×SA	12702.1**	< 0.001	6385.5**	< 0.001	350.4**	< 0.001	6485.9**	< 0.001	33.1**	< 0.001
D×SA	417.0**	< 0.001	543.2**	< 0.001	65.6**	< 0.001	490.0**	< 0.001	76.6**	< 0.001
V×D×SA	1007.8**	< 0.001	710.1**	< 0.001	44.8**	< 0.001	523.0**	< 0.001	34.5**	< 0.001

V—Cr(III), Cr (VI). D—dose of Cr. SA—soil amendments. V×D, V×SA, D×SA, V×D×SA—interactions between the factors. *Significant at *p* < 0.05; **significant at *p* < 0.01. ns—not significant

**Table 4:** Analysis of variance (*F*-test) of heavy-metal content in aboveground parts of maize

Source of variation	Cr		Cu		Zn		Ni		Biomass yield	
	*F*	*p* value	*F*	*p* value	*F*	*p* value	*F*	*p* value	*F*	*p* value
V	8959.2**	< 0.001	7562.8**	< 0.001	8345.4**	< 0.001	1401.96**	< 0.001	3479**	< 0.001
D	604.9**	< 0.001	136.3**	< 0.001	1199.0**	< 0.001	6493.05**	< 0.001	2066**	< 0.001
SA	2391.2**	< 0.001	240.7**	< 0.001	11070.6**	< 0.001	9492.36**	< 0.001	622**	< 0.001
V×D	721.7**	< 0.001	770.2**	< 0.001	564.5**	< 0.001	299.47**	< 0.001	2093**	< 0.001
V×SA	14104.5**	< 0.001	412.5**	< 0.001	3992.3**	< 0.001	1740.52**	< 0.001	3046**	< 0.001
D×SA	1014.6**	< 0.001	212.3**	< 0.001	442.6**	< 0.001	7073.30**	< 0.001	1831**	< 0.001
V×D×SA	995.3**	< 0.001	117.6**	< 0.001	619.4**	< 0.001	269.17**	< 0.001	1066**	< 0.001

V—Cr(III), Cr (VI). D—dose of Cr. SA—soil amendments. V×D, V×SA, D×SA, V×D×SA—interactions between the factors. *Significant at *p* < 0.05; **significant at *p* < 0.01. ns—not significant

**Fig. 2:**
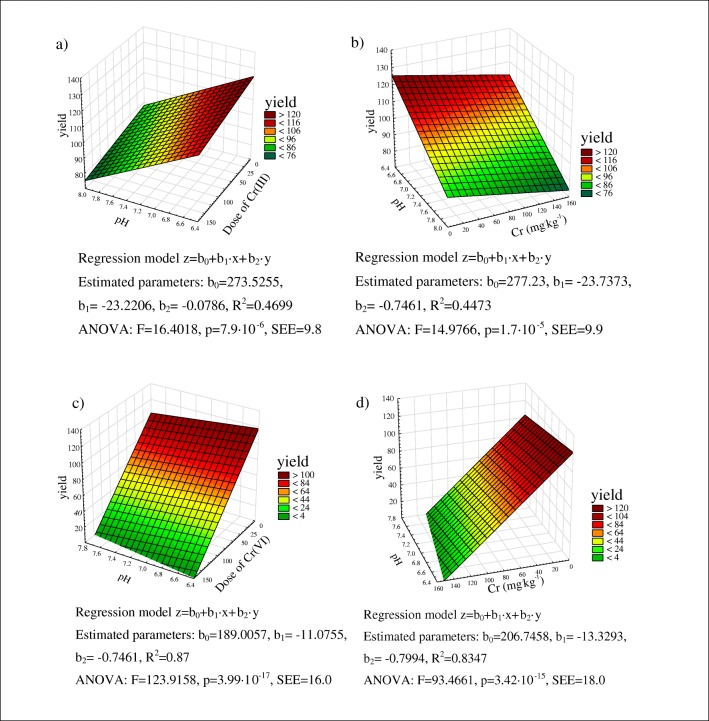
The relationship between spring barley biomass yield vs. Cr dose and soil pH. SEE, standard error of estimation

The multiple regression analysis of soil contamination with Cr(III) and Cr(VI) revealed that barley yields were influenced by Cr dose and soil pH in pots contaminated with Cr(III) and by soil pH in pots contaminated with Cr(VI). The spring barley model with *R*^2^ of 0.87 and standard error of estimation of 16.0 was characterized by the highest goodness of fit (Fig. 2). Maize yields were determined by soil pH, whereas the influence of Cr(III) and Cr(VI) doses was not significant (not shown).

### The effect of Cr(III) and Cr(VI) contamination on the heavy-metal content of plants

The Cr content of the aerial parts of spring barley (main crop) and maize (successive crop) was significantly affected by Cr(III) and Cr(VI) doses (Table 5). The average Cr content of the aerial parts of spring barley in pots without soil amendments was nearly twice higher in pots contaminated with Cr(III) than in pots contaminated with Cr(VI). The reverse was noted in maize (successive crop), where the Cr content of aerial plant parts was three times higher in pots contaminated with Cr(VI) than in Cr(III) treatments. Cr(III) exerted a greater influence on the Cr content of the aboveground biomass of spring barley, where Cr(III) doses of 100 and 150 mg kg^−1^ soil led to a 5-fold increase in Cr concentration relative to the control treatment. In treatments without soil amendments, an increase in Cr(III) and Cr(VI) doses increased the Cr content of the aerial biomass of maize. The increase was higher in pots contaminated with Cr(VI) where Cr concentration increased nearly 17-fold relative to the control treatment (without Cr).

**Table 5:** Effect of chromium contamination and soil amendments on heavy-metal content in spring barley (*Hordeum vulgare* L.) and maize (*Zea mays* L.), mg kg^−1^

Cr dose in mg kg^−1^ of soil	Kind of contamination															
	Cr(III)		Cr(VI)		Cr(III)		Cr(VI)		Cr(III)		Cr(VI)		Cr(III)		Cr(VI)	
	Kind of soil amendments															
	Without soil amendments		Without soil amendments		Compost		Compost		Zeolite		Zeolite		Calcium oxide		Calcium oxide	
Content of Cr (mg kg^−1^)																
Barley (*Hordeum vulgare* L.)—main crop																
0	1.42	a	1.42	a	0.55	a	0.55	a	1.55	a	1.55	a	2.37	b	2.37	b
25	3.65	d	1.73	b	1.17	c	0.60	a	1.27	a	7.37	d	1.47	a	6.27	d
50	3.82	d	1.73	b	1.25	cd	0.80	ab	1.28	a	6.03	c	1.25	a	7.18	e
100	4.70	e	2.85	c	1.12	bc	1.53	d	2.25	b	10.95	e	1.47	a	7.68	f
150	6.83	f	3.08	c	1.40	cd	3.03	e	2.42	b	11.02	e	2.82	c	8.30	g
Maize (*Zea mays* L.)—successive crop																
0	0.47	a	0.47	a	1.37	b	1.37	b	4.50	b	4.50	b	4.00	c	4.00	c
25	2.38	bc	7.05	d	2.77	e	2.47	d	4.98	c	0.72	a	5.58	d	0.65	a
v50	2.02	b	7.12	d	3.53	f	1.68	c	5.93	d	0.42	a	6.53	e	0.62	a
100	2.23	bc	7.27	d	3.58	f	0.48	a	4.17	b	0.53	a	7.43	f	0.78	a
150	2.55	c	7.90	e	4.07	g	0.50	a	6.20	d	0.58	a	7.52	f	1.35	b
Content of Cu (mg kg^−1^)																
Barley (*Hordeum vulgare* L.)—main crop																
0	2.82	a	2.82	a	5.03	a	5.03	a	2.10	bc	2.10	bc	2.23	a	2.23	a
25	2.97	ab	4.63	d	5.35	ab	5.58	ab	0.63	a	12.82	d	5.17	c	12.93	g
50	3.30	b	5.78	e	5.35	ab	12.58	d	0.98	a	14.90	e	5.57	cd	12.03	e
100	3.93	c	5.48	e	6.05	b	12.45	d	1.88	b	14.92	e	5.37	d	12.50	f
150	7.93	f	3.92	c	8.38	c	14.80	e	2.63	c	15.20	e	4.53	b	12.88	g
Maize (*Zea mays* L.)—successive crop																
0	10.05	a	10.05	a	12.03	c	12.03	c	14.23	c	14.23	c	11.63	c	11.63	c
25	15.38	e	14.43	d	11.32	bc	12.20	c	16.80	f	7.97	b	13.15	d	10.10	b
50	17.87	g	12.13	b	19.55	f	10.32	b	16.05	e	7.07	a	14.80	e	8.87	a
100	17.27	f	13.82	c	16.70	e	6.83	a	15.45	d	7.20	a	16.35	f	9.70	ab
150	12.15	b	13.67	c	13.97	d	7.68	a	14.52	c	7.77	b	14.57	e	9.87	ab
Content of Zn (mg kg^−1^)																
Barley (*Hordeum vulgare* L.)—main crop																
0	128.90	f	128.90	f	125.90	e	125.90	e	124.00	f	124.00	f	108.20	e	108.20	e
25	104.30	e	85.60	cd	114.10	d	93.20	b	121.00	def	88.00	c	99.40	d	77.80	b
50	89.30	d	80.00	bc	120.30	de	69.50	a	122.50	ef	67.70	a	95.80	cd	74.50	b
100	73.60	ab	72.60	a	102.10	c	65.20	a	115.50	d	72.50	ab	89.80	c	65.30	a
150	72.30	a	70.20	a	87.90	b	63.40	a	116.80	de	75.05	b	75.90	b	69.60	ab
Maize (*Zea mays* L.)—successive crop																
0	82.30	e	82.30	e	57.90	d	57.90	d	41.80	d	41.80	d	27.60	c	27.60	c
25	60.60	c	49.20	a	48.20	b	42.40	a	42.90	d	7.50	a	30.30	d	9.10	b
50	69.50	d	47.20	a	48.20	b	53.30	c	44.30	d	10.80	ab	40.60	d	5.00	a
100	67.50	d	47.60	a	48.90	b	68.20	e	48.60	e	11.60	bc	51.60	f	4.00	a
150	54.30	b	47.10	a	50.80	bc	99.50	f	69.30	f	14.50	c	51.40	f	2.70	a
Content of Ni (mg kg^−1^)																
Barley (*Hordeum vulgare* L.)—main crop																
0	20.30	e	20.30	e	19.72	g	19.72	g	14.23	d	14.23	d	17.95	c	17.95	c
25	9.60	d	6.03	c	15.18	f	5.00	a	9.37	bc	41.27	f	3.43	b	23.58	d
50	9.27	d	3.98	b	15.78	f	4.53	a	11.37	cd	25.90	e	1.92	a	28.17	f
100	8.68	d	2.30	a	12.18	e	6.82	b	7.10	ab	28.80	e	1.68	a	25.6	e
150	6.43	c	1.33	a	10.97	d	8.95	c	5.18	a	26.5	e	1.45	a	26.3	e
Maize (*Zea mays* L.)—successive crop																
0	61.43	f	61.43	f	5.50	b	5.50	b	4.07	c	4.07	c	2.10	bc	2.10	bc
25	16.33	e	5.17	c	25.55	f	5.58	b	1.17	a	6.63	f	1.70	a	4.15	g
50	13.80	d	1.37	a	14.87	e	2.47	a	1.17	a	5.37	e	2.22	c	3.20	e
100	4.45	bc	3.13	abc	11.53	d	2.13	a	1.23	a	5.03	d	1.93	ab	2.50	d
150	1.27	a	2.40	ab	7.90	c	2.17	a	1.20	a	3.37	b	3.10	e	3.63	f

Different letters indicate significant difference at *p* < 0.05

Both Cr(III) and Cr(VI) significantly affected the Cu content of the aerial parts of spring barley and maize (Table 5). In treatments without soil amendments, the average Cu content of the aboveground biomass of spring barley (main crop) was somewhat higher in Cr(VI) treatments than in Cr(III) treatments. In pots without soil amendments, the Cu content of the aerial parts of spring barley was positively correlated with increasing doses of Cr(III) which induced a nearly 3-fold increase in Cu concentration. Cr(III) exerted a greater influence on the Cu content of maize, and the Cr(III) dose of 100 mg kg^−1^ soil increased the analyzed parameter by 78% relative to the control treatment. Higher doses of Cr(III) decreased the Cu content of maize.

In treatments without soil amendments, the average Zn content of the aboveground parts of spring barley and maize was higher in pots contaminated with Cr(III) than in Cr(VI) treatments (Table 5). In treatments without soil amendments, Cr(VI) exerted a minimally greater influence on the Zn content of the aerial biomass of spring barley. The Cr(VI) dose of 150 mg kg^−1^ soil decreased Zn concentration in spring barley by 46% relative to the control treatment (without Cr). The accumulation of Zn in the aboveground parts of spring barley in treatments without soil amendments was negatively correlated with increasing doses of Cr(III). In these treatments, the Cr(III) dose of 150 mg kg^−1^ soil decreased Zn content by 44% relative to the control treatment (without Cr). In non-amended treatments contaminated with Cr(VI), the decrease in the Zn content of maize was similar to that noted in spring barley, whereas Cr(III) exerted a far smaller influence.

In treatments without soil amendments, the average Ni content of the aboveground parts of spring barley and maize was higher in pots contaminated with Cr(III) than in Cr(VI) treatments (Table 5). Cr(VI) exerted a greater influence on the Ni content of spring barley in non-amended treatments than Cr(III). The accumulation of Ni in the aerial biomass of spring barley (main crop) in non-amended treatments was negatively correlated with increasing doses of Cr(III) and Cr(VI). In these treatments, Cr(VI) doses of 100 and 150 mg kg^−1^ soil led to a nearly 9-fold and 15-fold decrease in Ni concentrations in the aerial parts of spring barley, respectively, relative to the control treatment (without Cr). Cr(III) caused an over 2-fold and 3-fold decrease in the Ni content of spring barley, respectively. Cr(VI) exerted a greater influence on maize (successive crop) than on spring barley. The highest dose of Cr(III) and Cr(IV)—150 mg kg^−1^ soil—decreased the Ni content of maize by 98% and 96%, respectively, relative to the control treatment (without Cr).

### The effect of soil amendments on dry biomass yield

The application of compost, zeolite, and calcium oxide in Cr(III) and Cr(VI) treatments did not improve crop yields. Soil amendments increased the average yield of the aerial biomass of maize only in pots contaminated with Cr(III) relative to the control treatment (Fig. 1). The application of calcium oxide in pots with the highest Cr(VI) dose of 150 mg kg^−1^ soil nearly completely inhibited the yielding of both crops.

### The effect of soil amendments on the heavy-metal content of plants

Compost, zeolite, and calcium oxide significantly affected the Cr content of the aerial parts of spring barley (main crop) and maize (successive crop) (Table 5). In treatments contaminated with Cr(III), all soil amendments decreased the average Cr concentration in the aboveground parts of spring barley. Compost was most effective (74%), whereas zeolite and calcium oxide (54%) were less-effective soil amendments. In pots contaminated with Cr(VI), only compost contributed to a 40% decrease in the Cr content of the aerial biomass of spring barley, whereas zeolite and calcium oxide increased Cr accumulation 3-fold and more than 2-fold, respectively, relative to the control treatment (without soil amendments). Compost (− 78%), zeolite (− 77%), and calcium oxide (− 75%) exerted the greatest influence on the Cr content of the aerial biomass of maize in treatments contaminated with Cr(VI), relative to the control treatment. In pots contaminated with Cr(III), all of the tested soil amendments increased Cr accumulation in maize, and the greatest increase was caused by calcium oxide and zeolite.

Compost, zeolite, and calcium oxide significantly influenced the Cu content of the aerial biomass of the tested crops in pots contaminated with Cr(III) and Cr(VI) (Table 5). In these treatments, compost, zeolite (only in Cr(VI) treatments), and calcium oxide increased the average Cr concentration in the aboveground parts of spring barley relative to the control treatment (without soil amendments). The addition of compost to pots with Cr(III) and the addition of zeolite to pots with Cr(VI) induced the highest increase in the Cu content of spring barley by 44% and 147%, respectively. The analyzed soil amendments exerted a greater influence on maize in treatments contaminated with Cr(VI) than Cr(III). In pots with Cr(VI), compost, calcium oxide, and zeolite decreased the Cu content of the aerial biomass of maize by 22–31% on average relative to the control treatment (without soil amendments). Soil amendments significantly modified Zn concentration in the aboveground parts of spring barley (main crop) and maize (successive crop) (Table 5). In treatments contaminated with Cr(III), the average Zn content of the aboveground parts of spring barley was most significantly increased by zeolite and compost relative to the control treatment (without soil amendments). The analyzed amendments were less effective in Cr(VI) treatments. In pots contaminated with Cr(III) and Cr(VI), calcium oxide and zeolite caused the greatest decrease in the average Zn content of maize (successive crop). However, the tested amendments exerted the greatest effect on the Zn content of maize contaminated with Cr(VI). Compost, zeolite, and calcium oxide had a significant influence on the Ni content of the aboveground parts of the tested crops (Table 5). The accumulation of Ni in the aerial biomass of spring barley was most significantly determined by compost (+ 36% on average) in Cr(III) treatments and by zeolite (+ 306%) and calcium oxide (+ 242%) in Cr(VI) treatments. In pots contaminated with Cr(VI), the high increase in Ni concentration under exposure to zeolite and calcium oxide can be attributed to the fact that average values were calculated only for pots with low doses of Cr(VI) due to the low availability of biomass samples from more contaminated treatments, which prevented the determination of Ni content. Compost enhanced the accumulation of Ni in spring barley in Cr(VI) treatments, whereas zeolite and, in particular, calcium oxide decreased the Ni content of spring barley in Cr(III) treatments. In treatments contaminated with Cr(III) and Cr(VI), all soil amendments decreased the Ni content of the aerial biomass of maize (successive crop) relative to the control treatment (without soil amendments). In pots with Cr(III), the greatest decrease in the Ni content of maize was induced by zeolite and calcium oxide at 91% and 89%, respectively, whereas compost and calcium oxide decreased Ni concentration in maize by 76% and 79%, respectively, in Cr(VI) treatments.

### The effect of Cr(III), Cr(VI), and soil amendments on soil pH

**Fig. 3:**
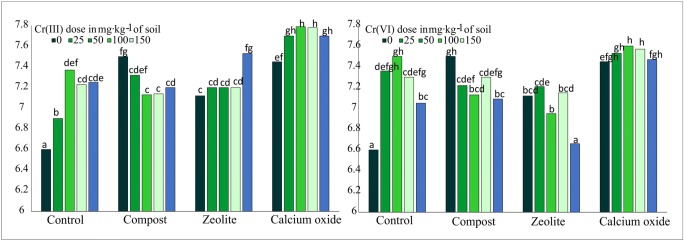
The pH value of soil in pots contaminated with different doses of Cr(III) and Cr(VI) (0; 25; 50; 150 mg kg^−1^ soil) and soil amendments. Different letters above the columns indicated significant difference at the *p <* 0.05

Contamination with Cr(III) and Cr(VI) significantly affected soil pH (Fig. 3). In treatments without soil amendments, small doses of Cr(III) and Cr(VI) soil (up to 50 mg kg^−1^) caused a gradual increase in soil pH, whereas higher Cr doses decreased the analyzed parameter. Soil contaminated with Cr(III) was characterized by slightly higher pH values. All of the evaluated soil amendments significantly influenced soil pH in spring barley and maize treatments contaminated with both Cr(III) and Cr(VI). Calcium oxide was most effective, and it induced a significant increase in soil pH relative to the control treatment. Zeolite and compost contributed to only a minor improvement in soil pH in treatments contaminated with Cr(III).

The effect of Cr(III) and Cr(VI) contamination on the heavy-metal content of soil

The average Cr content of soil was 4% higher in Cr(VI) treatments where spring barley and maize were grown without soil amendments (Table 6). In these pots, the highest Cr(VI) dose of 150 mg kg^−1^ soil induced an 11-fold increase in the Cr content of soil. A somewhat smaller, 10-fold increase was observed in Cr(III) treatments relative to the control treatment. After harvest, the average Cu content of soil was significantly higher in non-amended treatments exposed to Cr(III). In the control treatment (without amendments), a negative correlation was observed between increasing doses of Cr(III)and Cr(VI) and the Cu content of soil, where Cr(VI) exerted a far stronger effect. After harvest in non-amended treatments, the accumulation of Zn in soil was negatively correlated with increasing doses of Cr(III) and Cr(VI). In these treatments, the concentration of Zn was lowest in response to Cr(III) doses of 100 and 150 mg kg^−1^ soil. The Zn content of control soil was more than 10% lower. The average Ni content of soil in non-amended treatments after harvest was similar in pots contaminated with Cr(III) and Cr(VI). In these treatments, the concentration of Ni was negatively correlated with increasing doses of Cr(III) and Cr(VI), where Cr(VI) exerted a stronger effect on the studied parameter. However, the observed changes did not exceed several percent.

**Table 6:** Content of heavy metals in soil (mg kg^−1^)

Cr dose in mg kg^−1^ of soil	Kind of contamination															
	Cr(III)		Cr(VI)		Cr(III)		Cr(VI)		Cr(III)		Cr(VI)		Cr(III)		Cr(VI)	
	Kind of soil amendments															
	Without soil amendments		Without soil amendments		Compost		Compost		Zeolite		Zeolite		Calcium oxide		Calcium oxide	
Content of Cr (mg kg^−1^)																
0	13.18	a	13.18	a	14.65	a	14.65	a	11.85	a	11.85	a	11.45	a	11.45	a
25	24.67	c	18.30	b	24.14	c	19.05	b	18.65	b	17.15	b	18.20	b	19.00	b
50	45.56	d	48.15	d	47.65	d	45.45	d	48.40	c	48.15	c	47.80	d	41.40	c
100	95.47	e	97.90	e	90.45	e	94.35	e	81.40	d	86.50	e	90.20	e	88.45	e
150	133.22	f	147.36	g	128.35	f	146.90	g	124.10	f	132.85	g	143.20	f	146.95	f
Content of Cu (mg kg^−1^)																
0	24.67	de	24.67	de	24.06	g	24.06	g	20.10	a	20.10	a	19.59	f	19.59	f
25	24.91	e	18.69	b	23.86	ef	18.47	c	19.56	a	17.64	a	18.38	e	16.68	c
50	24.85	e	18.49	b	23.45	def	17.71	bc	18.57	a	16.83	a	18.17	de	16.25	bc
100	23.30	d	17.43	ab	22.95	de	17.12	ab	18.27	a	16.88	a	17.65	d	15.65	b
150	21.71	c	16.77	a	22.60	d	16.73	a	17.71	a	16.02	a	16.85	c	14.91	a
Content of Zn (mg kg^−1^)																
0	43.42	c	43.42	c	54.39	f	54.39	f	42.57	d	42.57	d	41.73	e	41.73	e
25	40.16	b	42.42	c	45.27	d	42.36	c	39.87	b	38.33	a	40.99	de	39.76	cd
50	38.24	ab	43.21	c	39.13	ab	41.85	bc	41.26	c	38.34	a	43.72	f	38.37	c
100	36.33	a	39.49	b	46.88	d	39.55	abc	40.27	bc	39.69	b	43.87	f	34.74	b
150	36.47	a	38.25	ab	49.82	e	38.24	a	40.32	bc	39.61	ab	45.02	f	30.75	a
Content of Ni (mg kg^−1^)																
0	5.74	e	5.74	e	3.32	b	3.32	b	3.38	ab	3.38	ab	2.56	b	2.56	b
25	5.56	e	4.60	d	2.88	a	2.65	a	3.65	bc	3.89	de	2.65	b	3.34	d
50	4.47	d	3.76	bc	2.83	a	2.65	a	3.12	a	4.06	de	2.67	b	3.04	c
100	4.20	cd	3.43	ab	2.83	a	3.27	b	3.63	bc	4.21	e	2.73	b	2.76	b
150	4.10	cd	3.02	a	2.75	a	3.93	c	4.06	de	5.11	f	3.23	cd	2.18	a

Different letters indicate significant difference at the *p* < 0.05

### The effect of soil amendments on the heavy-metal content of soil

In pots sown with spring barley and maize, all of the tested soil amendments decreased the average Cr content of soil in all experimental variants (Table 6). In treatments contaminated with Cr(III) and Cr(VI), the concentration of Cr was most effectively reduced by zeolite at 9% and 8%, respectively, relative to the control treatment. The Cu content of soil after harvest was reduced by all of the evaluated amendments in treatments contaminated with both Cr(III) and Cr(VI). Calcium oxide was most effective, and it decreased the concentration of Cu by 24% and 14%, respectively, relative to the control treatment (without soil amendments). The addition of compost increased the average Zn content of soil by 21% in Cr(III) treatments and by 5% in Cr(VI) treatments relative to the control treatment (without soil amendments). Calcium oxide had a weaker influence on Cr(III) and Cr(VI) treatments, and it exerted opposite effects on pots with Cr(III) and Cr(VI). All of the tested soil amendments negatively affected the average Ni content of soil in Cr(III) and Cr(VI) treatments after harvest. Calcium oxide and compost decreased the concentration of Ni by 43% and 39%, respectively, in Cr(III) treatments, and by 33% and 23%, respectively, in Cr(VI) treatments, relative to the control treatment (without soil amendments).

## Discussion

The results of the present study and literature data published in recent years (Wyszkowski and Radziemska 2009; Paul et al. 2015; Saleem et al. 2015; Μolla et al. 2017) indicate that Cr(III) and Cr(VI) compounds exert a negative effect on the natural environment, including soil and plants. Heavy metals accumulated in soil, including Cr, are easily transported to the aerial parts of plants, and they exert long-term effects on biotic components in ecosystems (Radziemska et al. 2017a). Assisted remediation decreases the availability of heavy metals and promotes the growth of permanent vegetative cover on contaminated land. In the presented study, spring barley (main crop) grown without soil amendments was highly sensitive to soil contamination with Cr(VI). In contrast, maize (successive crop) yields improved under exposure to increasing doses of Cr(III) and Cr(VI). Hexavalent chromium compounds are toxic for plants. When applied in identical doses, Cr_2_O_7_^2−^ is strongly toxic, whereas Cr(III) does not cause plant damage. Sensitive plants exhibit symptoms of toxicity already under exposure to a Cr(VI) dose of 1–2 mg kg^−1^ soil (Shahid et al. 2017). The changes observed in plants, such as reduced biomass yield, are indicative of the toxic effects of Cr compounds (Patra et al. 2018). Plants control heavy-metal concentrations by inhibiting their root uptake and transfer to tissues or by immobilizing contaminants through the formation of bonds with biologically active molecules (Hedayatkhaha et al. 2018).

In this study, the Cr content of the aerial parts of the tested plants increased with a rise in heavy-metal concentrations in soil. Our results are consistent with previous findings (Wyszkowski and Radziemska 2009, 2013; Sinha et al. 2018). The average concentrations of Cr, Zn, and Ni were higher in the aerial biomass of spring barley and maize exposed to Cr(III) than Cr(VI). Soil amendments that promote the mobilization or immobilization of soil contaminants play a key role in the process of removing or immobilizing Cr in soil and improving soil quality (Hamid et al. 2019). In a study by Antoniadis et al. (2018), the total content of Cr(III) and Cr(IV) in the aerial biomass of oregano reached 404.27 mg kg^−1^ DM in untreated soil and 423.33 mg kg^−1^ DM in soil treated with zeolite. Heavy metals can cause various changes in the soil ecosystem (Chu et al. 2018), and anthropogenic factors, including Cr contamination, significantly modify the physical, chemical, and biological properties of soil (Dotaniya et al. 2017). Soil parameters, in particular pH, granulometric composition, and the content of humic substances, significantly influence the oxidation of Cr compounds (He et al. 2018) and their toxic effects on plants. The leaching of base compounds from soil increased solubility of Mn and Al compounds and other phytotoxic substances, and a decrease in nutrient availability also altered the chemical properties of soil (Lee et al. 2019). Soil pH and organic matter content are the key determinants of the bioavailability of heavy metals and their effect on plants (Liu et al. 2019). The valence state and sorption of Cr in soil are affected by soil pH and redox potential. In soils with a pH of 5.5, Cr(III) cations are precipitated from the solution and, unlike Cr(VI) cations, are sparingly soluble. Cr(III) sorption increases, whereas Cr(VI) sorption decreases with a rise in soil acidity (Elouahli et al. 2018). In the present experiment, calcium oxide led to the greatest increase in soil pH relative to the control treatment. Zeolite and compost induced a smaller increase in soil pH in Cr(III) treatments.

Calcium oxide and, partly, zeolite also decreased the Cu and Ni content of soil. The results of this study and other authors’ findings indicate that zeolites effectively remove heavy metals, including Cr, from soil due to their high porosity and sorptive capacity (Antoniadis et al. 2018; Feng et al. 2018; Huang and Wei 2018). Eyvazi et al. (2019) found that nano-magnetic MnFe_2_O_4_ significantly enhanced the immobilization of Cr(VI) by decreasing leachability, plant bioavailability, human bioaccessibility, and risk of release. In comparison with other heavy metals, Cr is most strongly bound to soil organic matter (Hseu et al. 2018), which is why compost is an effective soil amendment (Adejumo et al. 2018; Goswami et al. 2018). In a greenhouse experiment conducted by Chen et al. (2018), compost decreased the availability of Cr by making it more stable and less mobile. Similar observations were made in this study, where the application of compost decreased the Cr content of soil in both experimental variants. In the presence of organic matter containing humic substances, Cr^3+^ ions are bound with or absorbed by surface-active organic and mineral compounds (Yin et al. 2018).

## Conclusions

Soil contamination with Cr is a major environmental and health concern. Spring barley (main crop) was highly sensitive to soil contamination with Cr(VI), whereas the biomass yield of maize (successive crop) increased with a rise in Cr(III) and Cr(VI) doses. The tested soil amendments increased the average yield of the aerial biomass of maize only in pots contaminated with Cr(III). In non-amended treatments, the average Cr, Zn, and Ni content of the aboveground parts of spring barley and maize was higher in pots with Cr(III) than in Cr(VI) treatments. The Cr content of maize was 3 times higher in Cr(VI) treatments than in Cr(III) treatments. The average concentration of Cu was higher in the aerial biomass of spring barley exposed to Cr(VI) than in that exposed to Cr(III). Calcium oxide significantly increased soil pH relative to the control treatment. In non-amended treatments, the average Cr content of soil was higher in Cr(VI) treatments. In these pots, the concentration of Cr increased 11-fold under exposure to the Cr(VI) dose of 150 mg kg^−1^ soil. In non-amended treatments, the accumulation of Zn and Cn was negatively correlated with increasing doses of Cr(III) and Cr(VI). Calcium oxide decreased the average content of Cr, Cu, and Ni in all experimental variants. The application of compost increased the average concentration of Zn in pots contaminated with Cr(III) and Cr(VI) relative to non-amended treatments.
